# The role of digital technologies in supporting quality improvement in Australian early childhood education and care settings

**DOI:** 10.1186/s40723-023-00107-6

**Published:** 2023-02-06

**Authors:** Maria Hatzigianni, Tanya Stephenson, Linda J. Harrison, Manjula Waniganayake, Philip Li, Lennie Barblett, Fay Hadley, Rebecca Andrews, Belinda Davis, Susan Irvine

**Affiliations:** 1 University of West Attica, Alsos Egaleo Campus, 28 St Spyridonos st., 11243 Athens, Greece; 2 Macquarie University, Sydney, Australia; 3 Edith Cowan University, Perth, Australia; 4 Queensland University of Technology, Brisbane, Australia

**Keywords:** Digital technology, Early childhood education, Quality improvement, Quality standards, Bronfenbrenner

## Abstract

This national study explored the role of digital technologies in early childhood education and care settings and whether they could contribute to quality improvement as reported by educators and assessors of quality in Australia. In this paper, data from Stage 2 of the Quality Improvement Research Project were used, which comprised 60 Quality Improvement Plans from educators linked with 60 Assessment and Rating reports from the assessors who visited early childhood centres as part of the administration of the National Quality Standards by each of Australia’s State and Territory jurisdictions. Bronfenbrenner’s ecological systems theory ( Bronfenbrenner, U. (1995). Developmental ecology through space and time: A future perspective. In P. Moen, G. H. Elder, Jr., & K. Lüscher (Eds.), Examining lives in context: Perspectives on the ecology of human development (pp. 619–647). American Psychological Association. 10.1037/10176-018; Bronfenbrenner & Ceci, Bronfenbrenner and Ceci, Psychological Review 101:568–586, 1994) was adopted to facilitate a systemic and dynamic view on the use of digital technologies in these 60 ECEC settings. References (e.g. comments/ suggestions/ examples) made by the educators about the implementation of digital technologies were counted and thematically analysed. Results revealed the strong role new technologies (e.g. documentation and management platforms, tablets, apps, etc.) play in the majority of ECEC settings and especially in relation to three of the seven Quality Areas: Educational programme and practice (Quality Area 1); Collaborative partnerships with families and communities (Quality Area 6) and Governance and leadership (Quality Area 7). Future directions for research are suggested and implications for embracing a more holistic, integrated and broad view on the use of digital technologies are discussed.

## Introduction

Continuous quality improvement is a key policy expectation of early childhood education and care (ECEC) settings in Australia (Australian Early Development Census [AEDC], 2019) and worldwide (Organisation for Economic Co-operation and Development [OECD], 2018). The Australian Government has been at the forefront of universal quality assessment and rating systems in the ECEC sector (Author et al., 2013; Tout et al., 2013). In Australia, ECEC caters for children birth to school entry and includes centre-based ECEC settings (e.g. preschool, kindergarten, long day care) and home-based family day care. The National Quality Framework (NQF) provides a national approach to quality assurance that integrates minimum quality standards as prescribed by law and aspirational quality standards to drive continuous quality improvement in education and care (ACECQA, 2021). To this effect, the NQF comprises a National Law and Regulations, a National Quality Standard (NQS), two Approved Learning Frameworks and a process for Assessment and Rating. Overseen by the Australian Children’s Education and Care Quality Authority (ACECQA), the day-to-day implementation of the NQF resides with State and Territory Regulatory Authorities. ACECQA “works with all governments to provide guidance, resources and services to support the sector to improve outcomes for children” (ACECQA, n.d.a, Table 6). The key responsibility here is the evaluation of ECEC setting quality against the seven quality areas of the NQS (ACECQA, HYPERLINK 2018, 2021). (Please note that the explanations of all acronyms are provided throughout this paper and in Table 6, Appendix B.)

The NQS evaluation process requires the ECEC setting to complete a self-assessment /evaluation of current practice and to submit a Quality Improvement Plan (QIP) (ACECQA, n.d.b) identifying perceived strengths and areas and strategies for continuous quality improvement. The QIP aims at helping “providers self-assess their performance in delivering quality education and care, and to plan future improvements” (ACECQA, n.d.b.).

A QIP must

- include an assessment of the programmes and practices at the service against the National Quality Standard and National Regulations;
- identify areas for improvement and
- include a statement about the service’s philosophy.

A QIP should also document and celebrate the service's strengths (ACECQA, n.d.b.). Subsequently, a trained assessor from the relevant State/Territory completes a visit to the ECEC setting, and using the A&R instrument (ACECQA, 2018) collects evidence to determine NQS scores against each of the seven Quality Areas (QA) (ACECQA, 2018; 2020):

- QA1: Educational programme and practice
- QA2: Children’s health and safety
- QA3: Physical environment
- QA4: Staffing arrangements
- QA5: Relationships with children
- QA6: Collaborative partnerships with families and communities
- QA7: Governance and leadership.

Following the visit, the assessor prepares a report for the Regulatory Authority, detailing evidence of practice and recommending ratings for each of these seven QAs (see ACECQA, n.d.c). Quality ratings are published and publicly available on a National Register. These ratings reflect the level of quality and are as follows:

1. *Exceeding* NQS: Service goes beyond the requirements of the National Quality Standard in at least four of the seven quality areas, with at least two of these being quality areas 1, 5, 6 or 7.
2. *Meeting* NQS: Service meets the National Quality Standard. Service provides quality education and care in all seven quality areas.
3. *Working Towards* NQS: Services provides a safe education and care programme. There are one or more areas identified for improvement.
4. *Significant Improvement Required*: Service does not meet one of the seven quality areas or a section of the legislation and there is a significant risk to the safety, health and wellbeing of children. The regulatory authority will take immediate action.

(For more details, see https://www.acecqa.gov.au/assessment/assessment-and-rating-process#quality%20ratings).

ECEC settings that achieve a rating of Exceeding NQS in all quality areas can apply, and offer evidence to be awarded an *Excellent* rating. For being awarded an Excellent rating by ACECQA (not the assessor) a service “promotes exceptional education and care, demonstrates sector leadership and is committed to continually improving” (https://www.acecqa.gov.au/assessment/excellent-rating). In August 2022, there were 38 ECEC services across Australia that had achieved an Excellent rating.

While data collected by ACECQA since the introduction of the NQS (ACECQA, 2012) have demonstrated improvement in areas such as programme and staffing quality, there are no specific elements or standards in the seven QAs for the role and impact of digital technologies in ECEC settings. This gap has become even more apparent in times of crisis, such as during the COVID-19 pandemic, where the key role of digital technologies in teaching and learning was required, but not guaranteed for everyone (e.g. digital divide became more evident for children, families and schools/centres with insufficient or no technological equipment, see studies by Chen, 2015; Dolan, 2016; Sosa Díaz, 2021).

This paper is based on data collected from 60 ECEC settings that participated in Stage 2 of a National Research project (the “Quality Improvement Research Project”), a sequential, three-phase investigation of ECEC settings that showed overall improvement in their NQS ratings over two consecutive A&R rounds (please see more details about the National Project in the Research Design section).

**Fig. 1:**
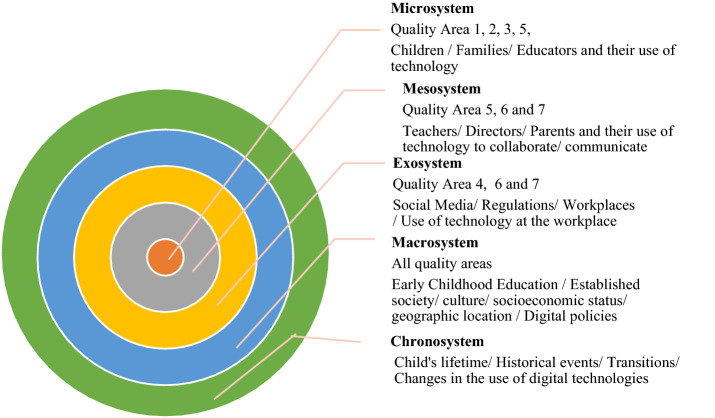
A visual representation of the main systems of the ecological theory and the alignment with the NQS Quality Areas and use of technology

In this study, we focused on the role of digital technologies in all areas related to ECEC and included in the NQS (e.g. the everyday programme; leadership; relationships; communication with parents; etc.). Early childhood teachers have made vital steps towards integrating the use of digital tools in their everyday practice (Dwyer et al., 2019). Networking, collaboration, communication with parents and documentation of children’s learning have been supported with the use of digital technologies (see, for example, James & Henry, 2017; McFaddden & Thomas, 2016; Parnell & Bartlett, 2012). Figure 1, adapted from Bronfenbrenner’s (1995) ecological systems theory demonstrates how technology when viewed systemically and holistically, may be embraced by ECEC stakeholders.

According to the ecological theory, all systems (nested circles: microsystem, mesosystem, exosystem, macrosystem and chronosystem) need to work harmoniously together so that they can contribute to optimise children’s learning and development. As depicted in Fig. 1, technology may be present in all the systems facilitating multidirectional relationships and interactions between the systems. Starting from inner circle of the microsystem, the everyday use of technology is evident in children’s homes and in early childhood settings (Donohue & Schomburg, 2017; Dorouka et al., 2020). The use of technology in the mesosystem for communication and collaboration purposes between parents, educators and other ECEC stakeholders is also widespread (McFadden & Thomas, 2016). In the outer systems of Exosystem and Macrosystem the presence of technology is again increasing rapidly though not sufficiently investigated (Murcia et al., 2018; Wright & Bales, 2014; Yost & Fan, 2014). Finally, the chronosystem refers to the current chronological period, the twenty-first century, a time of technological advancements. Therefore, technology can be seen as an organic, systemic element possibly present in all ECEC operations and processes and not as a standalone tool. If seen in such an organic, systemic way technology may contribute to the improvement of quality in ECEC by strengthening the relationships and balance between all systems. Approaching technology systemically has the potential to broaden our views around what technology entails. Seeing technology as a ‘process’ (Warschauer, 2002) or as “the underlying structure of our lives” (Castells, 2004, p. 224) will assist with understanding the role technology plays in our everyday lives, jobs and educational practices. This systemic approach will focus on a more dynamic, flexible, structural and organisational view of technology for ECEC sector (Keirl, 2015; Stevenson, 2008).

The following sections briefly review the research around the use of digital technologies in ECEC. The terms digital technology, digital or new technologies or technology are used interchangeably in this paper to include all kinds of digital devices, the internet, digital platforms, social media and any other technological resources (e.g. robotics, interactive whiteboards, etc.) which can be utilised for educational, communication, management, advertising or similar purposes in early childhood settings.

## Digital technologies in early childhood education and care

There has been an increasing use of technology in the field of education across all levels accompanied by a shift towards understanding which technologies can be used for specific purposes and exploring how best they can be used and embedded across educational contexts (Higgins et al., 2012). In ECEC, digital technology has been primarily used for pedagogical purposes, as a tool to support and advance the quality of teaching and learning in areas, such as literacy (e.g. Beschorner & Hutchison, 2013; Burnett, 2010; Neumann, 2018), mathematics (Cicconi, 2014; Sinclair, 2018) and STEM (e.g. Dorouka et al., 2020; Kermani & Aldemir, 2015; Marsh et al., 2018). There has also been an increasing focus on the pedagogical importance of introducing digital technologies in early childhood environments and the various opportunities and demands facing early childhood educators aiming to integrate digital technologies to encourage problem solving and computational thinking in young children (e.g. Bers et al., 2013, 2014; Murcia et al., 2018). As a result, there is a plethora of research available around the use of digital technologies in relation to Quality Area 1: Educational programme and practice (Beschorner & Hutchison, 2013; Burnett, 2010; Dorouka et al., 2020; Murcia et al., 2018; Sinclair, 2018) but limited research in relation to other areas of the NQS, such as Quality Area 5 (relationships with children, for example, Hooker, 2019; Kaplan-Berkley, 2022) and Quality Area 6 (Collaborative partnerships with families and communities, see, for example, da Silva et al., 2018; Higgins & Cherrington, 2017).

Digital technologies can also play a major role in supporting early childhood educators with planning and documentation. Careful selection of tools and applications that include an element of progress monitoring can be used to assist educators with consistent and systematic observations of children’s learning and development (Lyons & Tredwell, 2015). Technology tools, such as digital cameras, digital audio devices, scanners and electronic portfolios, can support educators to systematically monitor and document children’s progress, providing permanent records of children’s work (Hooker, 2015; Lyons & Tredwell, 2015). A variety of commercial web-based software are available to support the process of planning and documentation (Dwyer et al., 2019). Previous research suggests that educators are increasingly adopting such digital resources for the purpose of planning and documentation as it allows ECEC settings to streamline this process (Beaumont-Bates, 2017; da Silva & da Silva, 2018).

## Digital technologies and relationships with families and communities

A significant challenge faced by many early childhood educators is the lack of family involvement and the development of collaborative partnerships with families (Donahue & Schomburg, 2017; Willis & Exley, 2018). These partnerships are essential to develop strong relationships with children due to the vital role families play in their children’s education and care (Fan & Yost, 2019; Stamopoulos, 2018). ECEC settings commonly use digital technology to facilitate communication between educators and families, encouraging family participation in their children’s education (Beaumont-Bates, 2017; da Silva & da Silva, 2018; Dwyer et al., 2019). Commercial software provides a variety of ways to communicate with families, including learning story templates, conversations, video and audio (Beaumont-Bates, 2017). While social media integration in ECEC settings was not widely accepted in the past (Parette et al., 2010), its adoption has increased in recent years. There is now a greater use of short message settings, email and Facebook (Dwyer et al., 2019; Yost & Fan, 2014), as effective and efficient means for streamlining communication between staff and families (Yost & Fan, 2014).

The speed of communication via digital platforms provides staff and families with an efficient way to engage with each other, maintain connections and stay updated with information and events (Goodman & Cherrington, 2015; Yost & Fan, 2014). While some families prefer face-to-face communications due to issues of familiarity, layout, user friendliness and cultural barriers, including written and spoken English as a second language, parents and ECEC staff favour the immediacy of exchanging information in an online environment. Parents also favour the use of social media as it provides access to additional insights and details about their child's everyday routines (Donohue & Schomburg, 2017; Goodman & Cherrington, 2015; Stratigos & Fenech, 2021; Yost & Fan, 2014). However, there is limited research investigating the voices of early childhood educators on this issue, the impact on their workload and whether the various forms of communication facilitated by digital technologies has actually improved the quality of relationships, partnerships and parents’ input (Parnell & Bartlett, 2012; Stratigos & Fenech, 2021).

Another area where the advancement of technology had influenced ECEC settings is the transition from hardcopy portfolios to e-portfolios via online platforms (Beaumont-Bates, 2017; Hooker, 2015; Penman, 2014). E-portfolios seem to support and enhance the development of collaborative partnerships between educators, children and parents by enabling educators to draw on the families’ funds of knowledge more easily to support children’s learning and wellbeing. Parents have a better understanding of their child’s day due to the immediacy and accessibility of e-portfolios. Parents can share this with their wider family network who may reside in other countries and typically have little physical contact with their grandchild, niece or nephew (Beaumont-Bates, 2017; Hooker, 2015; Penman, 2014).

The issue of equal access and opportunities to take advantage of digital resources has been widely discussed during the COVID-19 pandemic (Flack et al., 2020; United Nations Association of Australia, 2021). Researchers in the field of digital technologies and early childhood have for many years signalled the need to eliminate the digital divide and take necessary measures (e.g. increase funding for early childhood education; provide both hardcopy and digital copies of important information) in order for all children and families to be able to benefit from digital affordances and not feel excluded (Daugherty et al., 2014; Dolan, 2016; Stamopoulos, 2018).

## Digital technologies and leadership in early childhood education and care

With the exception of professional development for educators, we found no empirical studies on leadership or types of leadership in association with digital technology in ECEC. Ongoing professional development is essential for early childhood professionals and digital technology has the potential to reduce associated costs and improve access to professional development resources (Ackerman, 2017; Donahue & Fox, 2012; Wright & Bales, 2014). Early childhood educators build their own professional knowledge through formal and informal online resources and have reported using a range of digital devices, with desktop computers being the most popular, followed by laptops, tablets and smartphones (Dwyer et al., 2019; Hatzigianni, 2018). For example, early childhood educators search for practical information, including ideas for activities and networking with other professionals through events, such as webinars and conferences or the use of a digital professional space to facilitate sharing of information, ideas and advice, and staying informed of industry updates (Dwyer et al., 2019). This highlights their professional learning needs can be met by the use of digital tools and the potential value of online learning (Donahue & Fox, 2012; Stone-MacDonald & Douglass, 2015; Wright & Bales, 2014).

Although the research around programme planning and learning with digital technologies has been steadily growing over the years, no research to our knowledge has examined the association of technology with the improvement of Governance and Leadership (QA7) in ECEC. More needs to be known about where and how digital technology is integrated in other areas of the ECEC context to understand how it can be used in supporting quality improvement.

Overall, as explained in the introduction section, this study is part of a larger National project and focused on answering two research questions:

1. How digital technologies are referred to the QIPs and A&R reports of ECEC services that demonstrated quality improvement to Meeting NQS and to Exceeding NQS?
2. How are digital technologies used and for what purposes in relation to the NQS Quality Areas?

## Research design

This study was part of a national study (2018) of quality improvement commissioned by ACECQA to identify the characteristics and drivers of quality improvement in centre-based long day care (LDC) settings (Harrison, 2019) that had improved their rating from Working Towards NQS at assessment Time 1 to Meeting NQS or Exceeding NQS at assessment Time 2 (please see explanation of the ratings in introduction). Three Australian universities were involved and a team of experts in this field worked collaboratively after gaining approval from their university human research ethics committees. This study consisted of three phases and followed a mixed methodology approach including quantitative analysis of a large dataset on 1338 LDC services accessed through the National Quality Agenda IT System 1 (Stage 1), qualitative analysis of a sub-sample of 60 LDC services’ QIPs and A&R reports (Stage 2) and in-depth examination 15 LDCs/case study centres (Stage 3).

This paper focuses on Stage 2, which applied proportional stratified random sampling to select 60 ECEC settings from the Stage 1 sample. Of these, 43 (72%) had improved from Working Towards NQS at Time 1 to Meeting NQS at Time 2, and 17 (28%) had improved from Working Towards NQS at Time 1 to Exceeding NQS at Time 2. As a group, the selected ECEC settings were representative of Australia’s eight States and Territories and five broad characteristics: Accessibility/Remoteness Index of Australia (ARIA +) categories (metropolitan, inner regional, outer regional, remote and very remote); community advantage/disadvantage (Socio-Economic Indexes for Areas [SEIFA] quintiles); management type (for-profit, not-for-profit); size of approved provider (small/standalone, medium, large); service size/number of approved places (< 60, ≥ 60) and no/transfer vs transfer of ownership.

The dataset comprised de-identified QIPs and A&R reports that were provided for these 60 LDC services by the Regulatory Authorities for each State and Territory. The sample distribution by jurisdiction is summarised in Table 1.

## Descriptive analysis and results

The data analysis began with a careful reading of all 120 documents (60 QIPs and 60 A7Rs). A list of technology related keywords, including technology, digital (dig*), electronic, computers, tablet/s, laptop/s, website/s and many others (see Table 2), was created after scanning the documents. A computer search was performed in all 120 documents to locate any relevant keywords from the list. A comprehensive Excel spreadsheet was created for each State/Territory with the seven Quality Areas. The researchers explored each document separately and added the references to technology verbatim.

The second step of the analysis involved careful reading and calculation of the references found under each Quality Area/jurisdiction/service. This resulted in the creation of a large table with the total number of references for each State/Territory per Quality Area (Appendix A) and two summary tables related to the centre’s overall quality rating (Tables 3 and 4). Out of all centres examined only two centres (from NSW) had zero references to digital technology (one Exceeding NQS and one Meeting NQS).

To examine possible differences between the ECEC settings that improved their rating from Working Towards NQS to Meeting NQS or Exceeding NQS, a descriptive analysis was conducted based on each centre’s quality rating (research question 1). The Exceeding NQS and Meeting NQS centres with the highest number of references to technology in each State/Territory jurisdiction were selected to facilitate comparisons and examine differences/similarities (8 Exceeding NQS + 8 Meeting NQS). Table 3 shows that Meeting NQS settings had more references to technology than the ones Exceeding NQS, in both the QIPs and the A&R reports. This finding is interesting but difficult to interpret as we do not have access to the QIP and A&R documents from the centres’ previous assessments or any knowledge of references to technology when these centres were rated at Working Towards NQS. In addition, the number of references might be smaller but the explanations provided by Exceeding centres were more extensive, as will be discussed later in this section.

Examining the reports more thoroughly and deeply (Table 4) indicated that overall, most of the ECEC settings (47 out of 60 or 78%) used a form of digital technology (e.g. digital platforms/social media/websites/online portals, etc.) in their everyday operations. Table 4 shows the types of technology located in the documents (after the keyword search) under four categories (e.g. Facebook is under social media). The first category “digital platforms” includes all the substantive tools which help educators with a large range of operations such as administrative (e.g. payrolls) and/or educational (e.g. programme planning, communicating with families, e-portfolios, etc.). Tools that do not provide a large range of choices are grouped under the category “apps /software”. Differences were apparent for Exceeding NQS compared to Meeting NQS centre. A higher proportion of Exceeding NQS centres (52%) reported the use of a digital platform compared to the centres that had improved to Meeting NQS (23%).

The Exceeding NQS centres used popular digital platforms (paid tools) more often (52%) than Meeting NQS centres (23%) and offered rich explanations on the use of digital technologies and the affordances they offered (e.g. outcomes from the Early Years Learning Framework ready to be inserted in their everyday planning, secure data sharing, teacher portfolios, enhancing professional learning and others) demonstrating their confidence and constructive capabilities with the integration of new technologies in their service. Social media (free tools) was more likely to be used by centres that improved to Meeting NQS (25%) compared to Exceeding NQS centres (11%).

## Thematic analysis and results

The keywords found (Table 2) in the 120 documents were separately reviewed following a thematic analysis approach (Braun & Clarke, 2012). The first phase was about familiarising oneself with the data. Our data were very rich and required careful reading and reviewing. After reading is complete, coding, segmenting and searching for themes began. The final phase was about reviewing, defining and organising themes. After analysis was complete, findings were substantiated with relevant quotations. Coding was first completed by two researchers. Each jurisdiction was examined separately at the start. A final list of themes was comprised after the examination of all jurisdictions. Themes were then presented, discussed and finalised with the whole team of 10 researchers.

The highest number of references to digital technology made by educators, educational leaders, directors or assessors emerged in three Quality Areas (Appendix A): QA1 (*n* = 74), QA6 (*n* = 111) and QA7 (*n* = 107). The themes for these three Quality Areas, including direct quotes derived from the QIPs, are presented next. Assessors’ comments are included where necessary.

### Validity

This study is based only on secondary, descriptive, qualitative data collected by a highly credible source, ACECQA, and not by individuals. ACECQA double checked the provision, anonymity and credibility of documents before sending them to researchers. Validity of this study is examined according to Maxwell’s (1992) five kinds of validity for qualitative research. Descriptive validity was ensured as documents (QIPs and A&R reports) were completed by ECEC settings or trained Assessors directly and not by the researchers. To further warrant accuracy, keywords around technology were explored electronically with the word search tool and not manually. Interpretive validity is high as the exact quotes and examples provided in the documents were counted and were not changed in any way. The present study did not suggest any theory or explanation of theory, so theoretical validity is not applicable to this study. Generalizability cannot be assumed for ECEC settings in Australia or elsewhere as the number of documents was small. Evaluative validity is also not applicable to this study as the researchers attempted to explore, describe and explain the data under investigation and not to make evaluations.

### Reliability

Two researchers examined the data and discussed coding, themes and findings to enhance reliability. Final findings were then scrutinised by a team of 10 researchers. As this was an exploratory study, frequencies were used to facilitate descriptive comparisons (e.g. between ECEC settings with different ratings; between the seven quality areas) and to help identify which digital tools are the most common in ECEC settings. However, frequencies were not enough to answer the research questions and thematic analysis was also utilised.

### QA1: Educational programme and practice

Quality Area 1 is focused on teaching, planning and assessing. Four main themes were generated in relation to technology, as presented below.

#### Digital documentation

Participating centres used new technologies such as commercial digital platforms in their planning; observations/assessments of learning and in developing children’s portfolios. Critical reflections completed by educators were also added in these platforms.

#### Communication with families and sharing of information

Technology was used to encourage feedback from families and incorporate their views in curriculum plans. In many centres, policies/philosophies were displayed in the foyer via screens/tablets, etc. As reported by a centre in the ACT, “Families have the opportunity to review and comment on policies that affect them. This process can be completed via email, displays at the centre or a conversation and later documented”. Another example is provided by a QLD setting: “Information about the service's operation, activities and experiences, upcoming events, and the kindergarten programme is provided to families on the service face book page, in notices at the office, through electronic correspondence and through brochures and information displayed at the service”.

#### Educators’ training and collaboration

Educators attended various webinars depending on their needs and interests and collaborated to form a holistic view on children’s development. Educators also used technology to document: ‘spontaneous learning and reflections on their teaching…mind maps to show their progression of projects and learning for children of all age groups’.

#### Children’s learning

Examples of how to use technology for enhancing learning were provided in the QIPs, such as ‘to implement STEM in EC’, ‘…child appropriate websites are accessed under direct supervision by educators’ and ‘the educator provided an iPad to support the children to research about insects. One quote summarises educators’ views on technology: ‘The future requires innovative thinkers and as early childhood educators it is our duty to assist the development of children’s innovative thinking. We do this through embedding problem solving, technology and scaffolding children’s ideas and supporting them into fruition’.

Overall, in this QA1, sharing information with families and on the assessment and planning cycle was emphasised, with less emphasis on educators’ critical reflection. The assessors most often summarised what was already described by the educators, positively commented on the use of digital tools for planning, learning and assessments (e.g. online platforms found in the setting or social media used). For example, “documentation within individual child portfolios, communication books, photos and digital photo screens, displays of children engaged in activities and experiences, and the service’s Facebook page were available and easily accessed by families”; or “educators record individual and group observations to analyse learning and development”. The service’s digital programme allows for individual files to be set up for each child. Photographs are saved in individual files and work samples are collected to support observations’.

### QA6: Collaborative partnerships with families and communities

This Quality Area is focused on partnerships and relationships with families and communities. The main themes arising from the analysis included the following:

#### Communication

A variety of digital media (e.g. social media, online tools/platforms, emails, websites, etc.) were employed to enhance and strengthen relationships and collaborations between the ECEC settings and families.

#### Access and sharing of information

Digital media were used for distributing enriching information (e.g. around Indigenous practices; cultural awareness; etc.) and ECEC setting events (e.g. excursions, incursions, open day). Technology was also used to facilitate the induction of new families (e.g. “Our orientation process is adapted to suit the individual needs of all families as some families simply want to begin care and in which case the child has their orientation and first day combined. In the case of this happening we ensure that we are supporting them through this transition as well as reporting to their families during their first day through our first day Kindy Hub report”).

#### Constructive feedback and input

Different digital media offered opportunities to families to provide their feedback on children’s learning (e.g. contributing to their e-portfolios) or on the ECEC setting’s decision making/policies/QIPs.

#### Improvement of the ECEC setting’s advertising of events and strengthening of community relationships

A number of settings (*n* = 11) commented on how they used new technologies to enhance their presence within the community and also enable community involvement.

Overall, it was evident in the analysis that the majority of these LDC centres (78%) used some form of digital tools, with emails, websites and social media being among their top preferences. LDC centres underlined the importance of embracing a range of digital and face-to-face strategies to efficiently communicate with parents and to “not alienate any families”. These 60 settings had invested in digital technologies, and educators provided strong evidence of their familiarity with the use of new forms of communication. They have also recognised its supplementary role and that the physical, more traditional way of communicating with families is irreplaceable. In line with these views, the assessors’ comments also supported the use of technology, e.g. “Current information about the service was provided to families in a variety of accessible formats through the parent handbook, service newsletters, displays at the service and through informal discussions with families at arrival and departure times. Correspondence was relayed via email or hard copy to suit the needs of individual families”.

### QA7: Governance and leadership

In this Quality Area, the focus was on effective leadership and management to improve the quality of the ECEC setting. References to QA7 were 107 in all documents (third most frequent references). The five themes emerging in this area were as follows:

#### Governance and management systems

ECEC settings provided ample information on how they displayed information; protected privacy; stored and secured documents and data. A range of technologies were available to support service operations including computers, printers, copiers and telephones, for example, “The service stored confidential information in locked filing cabinets located at the service, and information stored on computers was password protected”. A subtheme here was the ‘Information technology (IT) support’ provided by Exceeding ECEC settings and commented on very positively by the assessors (no negative comments were reported). Educators and/or directors viewed the existence of technical IT support helpful and reassuring for their everyday practice.

#### Communication with families and educators

Documents described which technological media and processes were adopted to facilitate the sharing of information with families, updating them on changes in policies/regulations and processes. Services also explained the way families provide feedback (e.g. through online surveys; USBs; digital platforms; tablets; etc.). Technology also helped educators communicate with each other and collaborate.

#### Online professional development for educators

The LDC staff used technology (e.g. digital platforms/ learning portals) to attend professional development (*n* = 40). With the help of technology, LDC services also organised successful online inductions for newly appointed educators (for example, a centre in SA reported: “The induction is for one day and includes online training in areas such as child protection and health and safety topics. All educators have their own email account and access to the intranet where relevant information is provided as well as online training and policy updates”).

This provision was also positively commented on by assessors. Assessors commented on the usefulness of digital tools for induction and professional development, for example, ‘The organisation has developed a comprehensive induction package, xxx, which is delivered across all services. This includes staff completing four hours of online training and a xxx workbook’ (xxx = identification name or similar which was removed by ACECQA to ensure anonymity and confidentiality); or ‘Management promote educators to stay up to date with current industry changes and educators regularly use ECA Learning hub and ECA blog posts and follow Multiverse Facebook page contributions by Dr … as part of this process’.

#### Improvement and the role of the educational leader:

The LDC documents referred to how technology can assist the centre with improving their practices and philosophy. For example, two centres in the ACT and TAS explained how the educators used their iPads to contribute to the writing of the QIP. The role of technology in assisting the work of the educational leader was also underlined. For example, in a centre in NSW, the aim for the educational leader was to ‘transfer the service to a paper free/digital programme’; other services offered suggestions on how technology was used by the educational leader to provide regular support and feedback to all educators (via emails/digital platforms/digital forums, etc.). Another service in QLD wrote: “The Educational Leader has developed a Professional Development room on Storypark, where professional development/training information is provided (to inspire and encourage attendance by the team), professional readings are provided. This can act as a provocation for professional conversation between colleagues and with the Ed[ucational] Leader. Storypark has also been adapted to include a First Australians”.

#### Sustainability

A small number (*n* = 7) of LDC services also referred to the need for becoming “paper free” to enhance sustainable practices through the adoption of digital practices.

## Discussion

This study explored the way educators and assessors referred to digital technologies and their use, implicitly through the completion of the Quality Improvement Plans (QIPs) and the Assessment and Rating (A&R) reports. Although these two documents do not explicitly address technology, the implementation and adjustment to the new digital era by ECEC settings were positively commented upon by the assessors in their reports (e.g. when the setting was using a digital platform for professional development or when a range of technologies was used for operational purposes). This study revealed a broad use of digital technologies with more frequent elaborations in the three Quality Areas: QA1: educational programme and practice; QA6: collaborative partnerships with family and community and QA7: Governance and leadership.

Within a systemic view, inspired by the Ecological theory (Bronfenbrenner, 1995; Bronfenbrenner & Ceci, 1994), the use of technology is embedded in all the systems—in the microsystem of designing and implementing programme planning and meaningful learning experiences for children; in the exosystem and mesosystem of communicating and collaborating with families and communities and in the mesosystem and macrosystem of updating policies, being informed about new laws and regulations and participating in professional development. This multidimensional view is consistent with the recommendations from the OECD (Slot, 2018) to explore the ECEC setting or organisational levels and the system or policy level of quality.

**Fig. 2:**
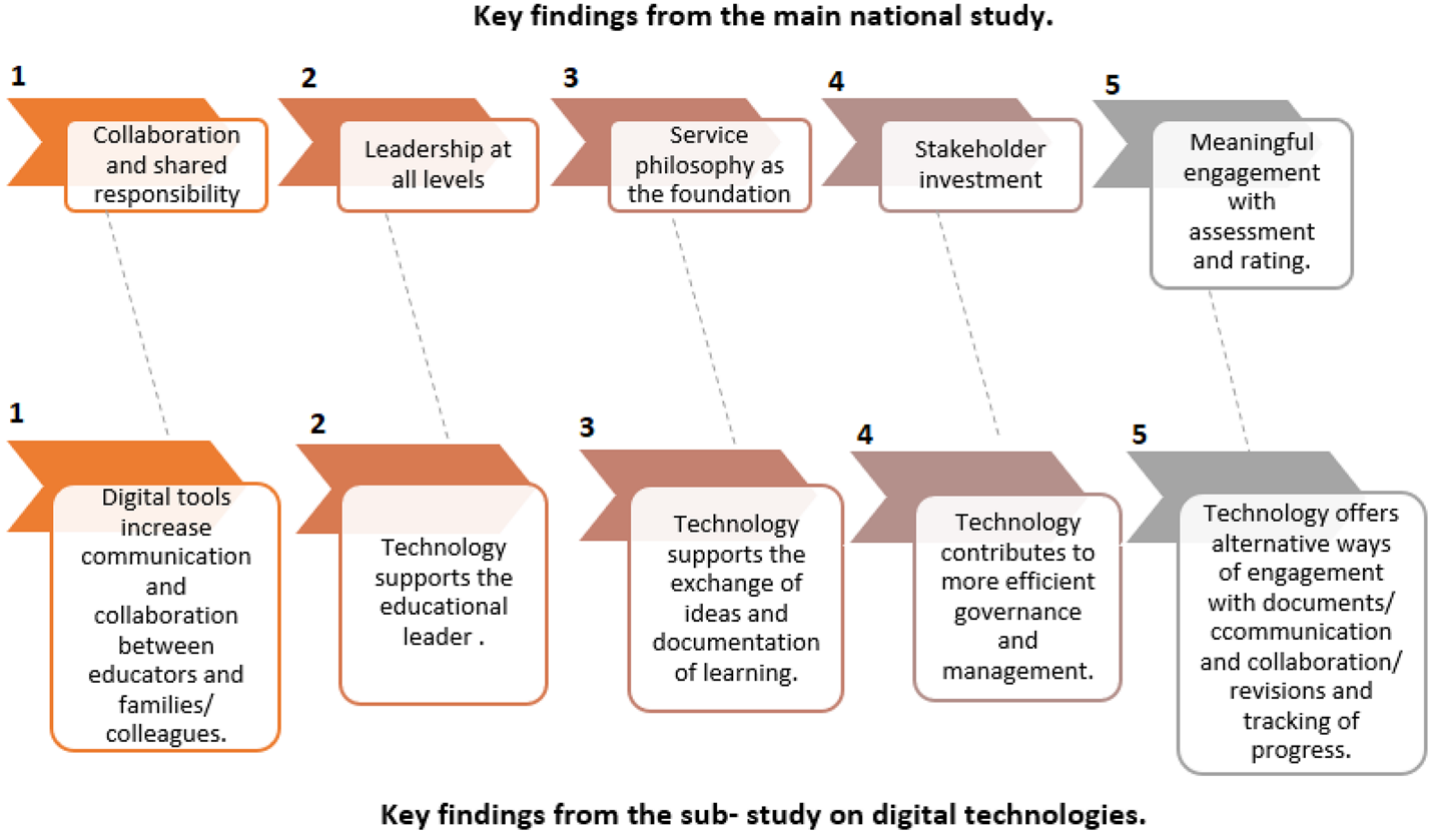
Aligning findings between the main national study and present sub-study on digital technologies

The strong role of digital technologies identified in the QIPs and A&R reports is in line with the key findings from the larger national project (Harrison, 2019) (see Fig. 2). Figure 2 presents the five key findings from the national study (on top) and how they are aligned with the references to the use of digital technologies examined in the present study. The first top key finding refers to “collaboration and shared responsibility” as a contributing factor to the improvement of quality for ECEC settings and is linked (dotted line) with ‘digital tools increase communication and collaboration between educators and families/colleagues’ (see thematic analysis section, themes ‘b’ and ‘c’ under QA1 and themes ‘a’ and ‘b’ under QA6).

The second key finding, leadership, was also found to be a decisive factor in enhancing quality and this study found that technology could support the work of the educational leader (see theme ‘d’ under QA7). However, there was no specific reference to how technology can support the roles and responsibilities of educators or educational leaders by the assessors. Assessors referred to the use of technology mostly for operational purposes, for example, “children were signed in and out of the service on a tablet computer using Kiosk and paper rolls (attendance) were also completed by educators in each room as children arrived and departed” and again there was not mention to whether technology could assist the work of educational leaders. Evidence of associations with educational leadership was not as frequent and was not sufficiently explained; this is worth the attention of future research. The challenge will be to uncover links between innovative technologies and strategic, empowering, collective leadership and decision making in the early years (James & Henry, 2017; Vanover & Hodges, 2015). More research in this field is imperative. For example, the advent and rapid growth of artificial intelligence has already impacted significantly the functioning and prospects of other sectors (e.g. in health care, science, engineering, etc.) (Bhatia et al., 2020; Chen et al., 2020; Roll & Wylie, 2016; Su & Yang, 2022). Questions of how artificial intelligence could support the quality of all levels of education will be of great interest and significance in the years to come (Su & Yang, 2022). A similar challenge would need to be addressed in relation to educational leadership. Research might need to re-examine the characteristics and skills of an effective, democratic and ethical leader in relation to their digital competences and decision making. The role of different forms of leadership (e.g. distributed leadership, transformational leadership, etc.) in encouraging or not the use of digital technologies would also be vital when investigating the barriers and enablers of integrating technology in everyday practice.

In addition, an area of fruitful research could also be whether digital tools could assist educational leaders’ pedagogical work. As explained by (Stamopoulos, 2018) a critical element of early childhood teachers’ professional practice is to ensure digital technologies are actually enhancing children’s learning.

A clear pedagogical philosophy (third key finding) was also considered to be an important quality factor in the national study. The findings of this study revealed that educators used a range of digital tools for pedagogical documentation and for making children’s everyday learning visible to families (see themes ‘a’ and ‘d’ under QA1). Research in this area is emerging (Stratigos & Fenech, 2021), indicating benefits, such as fast, easy access from any location; efficiency; extending learning from home; pedagogical benefits; richer insights and links with curriculum, theory and research and actively engaging children in the documentation process. Challenges have also been reported, including data security and privacy concerns, equity, increased workloads for educators, as well as decreasing interactions with children and other stakeholders (Higgins & Cherrington, 2017; Hooker, 2019; McFadden & Thomas, 2016; Penman, 2014; Plumb & Kautz, 2014). This line of research will need to grow in the future to better inform educators, parents, communities and other stakeholders on how to best design, select and use digital tools, addressing concerns and ensuring not only children’s learning but also a work-life balance and a sustainable and efficient role for early childhood professionals. A challenge for this line of research would be on one hand the rapidness of changing technology and on the other hand the lack of consistent, effective professional learning for early childhood educators (Donohue & Fox, 2012; Stone-MacDonald & Douglass, 2015).

The fourth key finding of the national study refers to ‘stakeholder investment’ and in this study the use of technology in governance and management was evident (see themes ‘a’, ‘b’, ‘c’, ‘e’ under QA7). E-governance practices, utilisation of management information systems and achieving inter-operability are new areas, and ongoing professional learning will be necessary for preservice and in-service educators (Bassok et al., 2019; Saitis & Saiti, 2018). Digital technologies can support the innovation and quality upgrade of the administrative and operational settings provided in ECEC settings.

Finally, the fifth key finding of the national study mentions “meaningful engagement with assessment and rating” as a way to increase quality for an ECEC setting. The role of digital tools to assist with this task can be very helpful. The use of online platforms, apps, software, social media and other tools could enhance flexibility and collaboration between educators of the same setting but also between educators of different settings to exchange views, exemplary practices and to support networking (see themes ‘b’, ‘c’, ‘d’ under QA7). However, more research is necessary in this field. Currently research has focused on digital tools and their impact on children’s learning and development and not on how digital technologies can improve administration, management and leadership practices in early childhood education (Abdullahi et al., 2019; Lindeman et al., 2021).

Overall, the 120 documents from 60 ECEC centres were randomly selected and meticulously examined. Although the sample is not representative of all the ECEC settings rated as Meeting or Exceeding in Australia, it is representative of the ECEC settings which made an improvement from Working Towards NQS and gained a higher rating. As an initial, exploratory attempt on examining the role of technology in quality improvement in ECEC settings, this study provides a useful basis for larger, future studies to build on. Challenges, such as insufficient and inconsistent professional development for early childhood educators in digital technologies (Hatzigianni, 2018; Romero-Tena, 2020; Thorpe et al., 2015; Tondeur et al., 2017), lack of resources and digital divide (Dolan, 2016; Sosa Díaz, 2021) are of primary concern when examining the integration of digital technologies and designing future research.

The researchers also recognise that their findings are only based on documents and reports, secondary data. Views of educators, assessors, managers and families were not directly examined and they would be critical in future studies. The researchers consider important to clarify that solely the use of technology does not improve quality or ratings of an ECEC setting. Although the findings of this study are to an extent encouraging for the use of digital technologies in early childhood education, improving quality is a much more complex endeavour which cannot be achieved just with the use of innovative tools (Tout, 2013; Yazejian & Iruka, 2015).

In comparing the number of references to digital technology by centre’s rating we found that the numbers were higher in Meeting NQS ECEC settings and that Exceeding NQS settings trusted commercial digital platforms more frequently. Although it is positive that settings from both ratings used technology, future research could examine specifically the type of digital affordances required or recommended for further improving quality and limiting concerns of both educators and parents.

Finally, this study also raises new directions for research, such as the provision of training, feedback and technological knowledge base of the NQS assessors. Offering advice, guidance and recommendations around the use of digital technologies in all Quality Areas could be helpful, together with the inclusion of relevant examples in the explanation of standards and elements in all Quality Areas. Additionally, the provisions around digital technologies in ECEC settings and educators’ professional learning need to be thoroughly examined in order for more equity and enhanced quality to be ensured. In Europe, sponsored by the European Commission (n.d.), an effort to improve the way, schools use technologies for teaching and learning, started in 2017 with SELFIE (Self-reflection on Effective Learning by Fostering the use of Innovative Educational technologies). The same tool is now being prepared for early childhood education educators. Something similar could be created in Australia and in other countries. A free tool, customisable, easy to use and adapt to different social contexts could be helpful for improving teaching and learning, maximising the digital potential of ECEC settings, assist with monitoring digital growth and minimising digital divide where needed.

## Conclusion

This study argues that this is the proper time to replace older, unidimensional views on technology with an organic, holistic view which is embedded in all areas of early childhood education. The role of technology is complex and dynamic, not static and passive. Realising the unquestionable role technology plays in many areas of everyday educational practice as a significant element of our pedagogical and educational philosophy will strengthen our understanding and enrich the ways technology is embedded in high-quality programmes.

Future research is essential in relation to communication, documentation and leadership. Although communication with parents and communities has already attracted research attention, more work needs to be completed in revealing the role, the availability and also possible limitations of digital tools for all involved. Similarly, digital documentation is an emerging field which requires more empirical evidence on whether or not it has a positive impact on children’s learning. Finally, the different types of leadership and whether they promote the integration of technology are a new line of research which requires careful consideration.

**Table 1:** Total number of documents analysed per State/Territory

State	No. of centres	No. of QIPs	No. of A&Rs
Australian Capital Territory (ACT)	4	4	4
Victoria (VIC)	8	8	8
Western Australia (WA)	8	8	8
Northern Territory (NT)	4	4	4
South Australia (SA)	5	5	5
Tasmania (TAS)	4	4	4
Queensland (QLD)	8	8	8
New South Wales (NSW)	19	19	19
Total	60	60	60

**Table 2:** Keyword Search Terms and Frequency distribution across the country

State/Frequency	WA	ACT	VIC	NSW	QLD	NT	TAS	SA	Total
Email	72	23	60	93	64	17	9	20	358
Online	34	13	15	32	28	13	3	12	150
Web(site)(inar)	10	17	6	32	28	6	4	12	115
Storypark	15	29	16	0	25	4	0	13	102
Computer	16	3	5	8	24	0	2	10	68
Elec(tronic)	5	8	2	17	16	1	2	1	52
iPad	3	6	4	20	7	0	3	5	48
Tech(nology)	2	2	1	17	14	4	2	2	44
Kindyhub (Kindy hub)	20	0	4	20	0	0	0	0	44
Facebook	4	2	4	9	9	2	7	6	43
Media	4	2	1	11	9	1	4	7	39
Digital	2	9	0	16	1	0	0	1	29
App(lication)	7	0	3	11	2	3	0	1	27
Platform	6	11	0	2	3	3	0	0	25
Early works	0	16	0	0	0	0	0	0	16
Internet	1	1	2	2	3	3	0	1	13
Educa	0	0	0	0	0	0	11	0	11
Laptop	3	0	0	5	0	1	0	0	9
Seesaw	4	0	0	0	0	0	0	0	4
Jigsaw	3	0	0	0	0	0	0	0	3
Qik Kids	0	0	0	0	0	1	0	1	2
eBook	0	0	0	0	0	0	0	0	0

**Table 3:** Number of references to technology by ECEC setting in each of the eight States/Territory (*N* = 16)

States	Exceeding NQS			Meeting NQS		
	QIP	A&R	Total	QIP	A&R A&R	Total
ACT	15	16	31	13	9	22
VIC	11	4	15	18	5	23
WA	13	7	20	18	8	26
NT	7	2	9	8	20	28
SA	12	12	24	10	9	19
TAS	8	6	14	14	8	22
QLD	11	17	28	14	8	22
NSW	19	0	19	16	15	31
Total For all states	96	64	160	111	82	193

**Table 4:** Kinds of digital tools used by centres as reported in the QIPs

	Exceeding centres (*n* = 17)	Meeting centres (*n *= 43)
Use of digital platforms (e.g. Storypark)	9 (52%)	10 (23%)
Social media (e.g. Facebook)	2 (11%)	11 (25%)
Websites/Blogs	2 (11%)	7 (16%)
Online software/apps/e-portfolios (e.g. Seesaw)	0	6 (13%)
Total	13 (77%)	34 (79%)

## Data Availability

Not applicable.

## Appendix A

See Table 5.

**Table 5:** Overview of number of references to technology per State/Territory

Area	ACT		NSW		VIC		QLD		WA		NT		TAS		SA		Total	
	Q	AR	Q	AR	Q	AR	Q	AR	Q	AR	Q	AR	Q	AR	Q	AR	Q	AR
QA1	22	10	50	24	24	9	16	16	14	3	6	3	3	2	5	7	140	74
QA2	7	6	17	6	8	1	7	5	13	7	2	0	1	0	1	4	56	29
QA3	2	0	10	2	3	2	7	10	3	2	0	1	0	1	3	2	28	20
QA4	3	3	12	5	3	1	7	5	7	2	3	0	0	0	2	2	37	18
QA5	0	0	4	0	0	0	2	0	1	0	1	0	2	0	3	1	13	1
QA6	16	10	39	23	12	13	15	24	20	14	5	7	6	7	4	13	117	111
QA7	5	14	30	24	6	5	7	23	10	12	2	10	3	5	4	14	67	107
Total	55	43	**162**	**84**	56	31	61	83	68	40	19	21	15	15	22	43	**458**	**360**
Total	98		24		87		144		108		40		30		65		818	
Q + AR	[8 docs]		[38 docs]		[16 docs]		[16 docs]		[16 docs]		[8 docs]		[8 docs]		[10 docs]		[120 docs]	

NSW - to show the highest number of all Australian States/Territories.

Total = to show the total number for all states.

## Appendix B

See Table 6.

**Table 6:** Explanation of the terminology used in this paper

Term	Abbreviation	Explanation (as stated at ACECQA’S website)
Australian Children's Education & Care Quality Authority	ACECQA	Works with all governments to provide guidance, resources and services to support the sector to improve outcomes for children
National Quality Framework	NQF	Provides a national approach to regulation, assessment and quality improvement for early childhood education and care and outside school hours care services across Australia
National Quality Standard	NQS	Ssets a high national benchmark for early childhood education and care and outside school hours care services in Australia
Quality Area	QA	The 7 quality areas of the NQS (QA1: Educational programme and practice, QA2: Children’s health and safety, QA3: Physical environment, QA4: Staffing arrangements, QA5: Relationships with children, QA6: Collaborative partnerships with families and communities, QA7: Governance and leadership
Quality Improvement Plan	QIP	The aim of a QIP is to help providers self-assess their performance in delivering quality education and care and to plan future improvements A QIP must: •Include an assessment of the programmes and practices at the service against the National Quality Standard and National Regulations; •Identify areas for improvement; •Include a statement about the service’s philosophy A QIP should also document and celebrate the service's strengths
Assessment and Rating report	A&R	Is a written record of the discussions and recommendations that happened during the service assessment
Early Years Learning Framework	EYLF	The National Framework for early childhood education in Australia

## References

[CR1] Abdullahi NJK, Adebayo TA (2019). Digitization in education system and management of early childhood care education in Nigeria. Southeast Asia Early Childhood.

[CR2] Ackerman DJ (2017). Online child care training in the United States: A preliminary investigation of who participates, what is offered, and on which topics the workforce is focusing. International Journal of Child Care and Education Policy.

[CR3] Australian Children’s Education and Care Quality Authority (ACECQA). (2012). *National quality framework for early childhood education and care.* ACECQA. https://www.acecqa.gov.au/national-quality-framework

[CR4] Australian Children’s Education and Care Quality Authority (ACECQA). (2018). *Quality rating reassessments. An analysis of quality improvement in education and care settings 2013–201. Occasional Paper 6. *https://www.acecqa.gov.au/sites/default/files/2018-07/OccasionalPaper6_QualityRatingReassessments.pdf

[CR5] Australian Children’s Education and Care Quality Authority (ACECQA). (2020). *Guide to the National quality framework*. https://www.acecqa.gov.au/sites/default/files/2022-03/Guide-to-the-NQFcompressed.pdf

[CR6] Australian Children’s Education and Care Quality Authority (ACECQA). (2021). *What is the NQF?* https://www.acecqa.gov.au/nqf/about

[CR7] Australian Children’s Education and Care Quality Authority (ACECQA). (n.d.a). *We support all governments and the education and care sector to realise the benefits of the National Quality Framework.* https://www.acecqa.gov.au/

[CR8] Australian Children’s Education and Care Quality Authority (ACECQA). (n.d.b). *Quality improvement plan*. https://www.acecqa.gov.au/assessment/quality-improvement-plans

[CR9] Australian Children’s Education and Care Quality Authority (ACECQA). (n.d.c). *Assessment and rating process.* https://www.acecqa.gov.au/assessment/assessment-and-rating-process

[CR10] Australian Early Development Census. (2019). *Quality improvement and policy.* https://www.aedc.gov.au/early-childhood/quality-improvement-and-policy

[CR11] Bassok D, Dee T, Latham S (2019). The effects of accountability incentives in early childhood education. Journal of Policy Analysis and Management.

[CR12] Beaumont-Bates JR (2017). E-Portfolios: Supporting collaborative partnerships in an early childhood center in Aotearoa/New Zealand. New Zealand Journal of Educational Studies.

[CR13] Bers MU, Flannery L, Kazakoff ER, Sullivan A (2014). Computational thinking and tinkering: Exploration of an early childhood robotics curriculum. Computers & Education.

[CR14] Bers M, Seddighin S, Sullivan A (2013). Ready for robotics: Bringing together the T and E of STEM in early childhood teacher education. Journal of Technology and Teacher Education.

[CR15] Beschorner B, Hutchison AC (2013). iPads as a literacy teaching tool in early childhood. International Journal of Education in Mathematics, Science and Technology.

[CR16] Bhatia N, Trivedi H, Safdar N, Heilbrun ME (2020). Artificial intelligence in quality improvement: Reviewing uses of artificial intelligence in noninterpretative processes from clinical decision support to education and feedback. Journal of the American College of Radiology.

[CR17] Braun V, Clarke V, Cooper H, Camic PM, Long DL, Panter AT, Rindskopf D, Sher KJ (2012). Thematic analysis. APA handbook of research methods in psychology. Research designs: Quantitative, qualitative, neuropsychological, and biological.

[CR18] Bronfenbrenner U, Moen P, Elder GH, Lüscher K (1995). Developmental ecology through space and time: A future perspective. Examining lives in context: Perspectives on the ecology of human development.

[CR19] Bronfenbrenner U, Ceci SJ (1994). Nature-nurture reconceptualized in developmental perspective: A bioecological model. Psychological Review.

[CR20] Burnett C (2010). Technology and literacy in early childhood educational settings: A review of research. Journal of Early Childhood Literacy.

[CR21] Castells M, McCarthy H, Miller P, Skidmore P (2004). Afterword: Why networks matter. Network logic: Who governs in an interconnected world?.

[CR22] Chen B (2015). Exploring the digital divide: The use of digital technologies in Ontario public schools. Canadian Journal of Learning and Technology.

[CR23] Chen L, Chen P, Lin Z (2020). Artificial intelligence in education: A review. IEEE Access.

[CR24] Cicconi M (2014). Vygotsky meets technology: A reinvention of collaboration in the early childhood mathematics classroom. Early Childhood Education Journal.

[CR26] da Silva YCA, da Silva MF (2018). Technological tools as a facilitator for parental/guardian participation in early childhood education. Nuevas Ideas En Informática Educativa [new Ideas in Information Education].

[CR27] Daugherty L, Dossani R, Johnson EE, Oguz M (2014). Using early childhood education to bridge the digital divide. Perspectives.

[CR28] Dolan JE (2016). Splicing the divide: A review of research on the evolving digital divide among K–12 students. Journal of Research on Technology in Education.

[CR29] Donohue C, Fox S (2012). Lessons learned, innovative practices, and emerging trends: Technology for teacher education and professional development. Exchange: the Early Childhood Leaders' Magazine.

[CR30] Donohue C, Schomburg R (2017). Technology and interactive media in early childhood programs: What we’ve learned from five years of research, policy, and practice. Young Children.

[CR31] Dorouka P, Papadakis S, Kalogiannakis M (2020). Tablets and apps for promoting robotics, mathematics, STEM education and literacy in early childhood education. International Journal of Mobile Learning and Organisation.

[CR32] Dwyer A, Jones C, Rosas L (2019). What digital technology do early childhood educators use and what digital resources do they seek?. Australasian Journal of Early Childhood.

[CR33] European Commission. (n.d). *SELFIE. How can your school improve how it uses technology for teaching and learning?* https://ec.europa.eu/education/schools-go-digital_en

[CR34] Flack, C. B., Walker, L., Bickerstaff, A., & Margetts, C. (2020). *Socioeconomic disparities in Australian schooling during the COVID-19 pandemic.* Pivot Professional Learning. https://pivotpl.com/wp-content/uploads/2020/07/Pivot_Socioeconomic-disparities-in-Australian-schooling-during-COVID-19_1July2020.pdf

[CR35] Fan S, Yost H (2019). Keeping connected: Exploring the potential of social media as a new avenue for communication and collaboration in early childhood education. International Journal of Early Years Education.

[CR36] Goodman N, Cherrington S (2015). Parent, whānau and teacher engagement through online portfolios in early childhood education. Early Childhood Folio.

[CR37] James F, Henry SF (2017). Building collective leadership capacity using collaborative twenty-first century digital tools. School Leadership & Management.

[CR38] Harrison, L. J., Hadley, F., Irvine, S., Davis, B., Barblett, L., Hatzigianni, M., Mulhearn, G., Waniganayake, M., Andrews, R., & Li, P. (2019). Quality Improvement Research Project. Sydney: Australian Children's Education and Care Quality Authority (ACECQA). 35 p. https://www.acecqa.gov.au/sites/default/files/2020-05/quality-improvement-research-project-2019.PDF

[CR39] Hatzigianni, M., & Kalaitzidis, I. (2018). Early childhood educators’ attitudes and beliefs around the use of touchscreen technologies by children under three years of age. *British Journal of Educational Technology*, *49*(5), 883–895.

[CR40] Higgins A, Cherrington S (2017). What’s the story? Exploring parent–educator communication through ePortfolios. Australasian Journal of Early Childhood.

[CR41] Higgins, S., Xiao, Z., & Katsipataki, M. (2012). *The impact of digital technology on learning: A summary for the Education Endowment Foundation*. https://www.semanticscholar.org/paper/The-Impact-of-Digital-Technology-on-Learning-%3A-A-Higgins-Xiao/d26bb59f2536107b57f242b8289b1eb6f51d8765

[CR42] Hooker T (2015). Assessment for learning: A comparative study of paper-based portfolios and online ePortfolios. Early Childhood Folio.

[CR43] Hooker T (2019). Using ePortfolios in early childhood education: Recalling, reconnecting, restarting and learning. Journal of Early Childhood Research.

[CR44] Irvine, S. & Farrell, A. (2013). The rise of government in early childhood education and care following the Child Care Act 1972: The lasting legacy of the 1990s in setting the reform agenda for ECEC in Australia. *Australasian Journal of Early Childhood*, *38*(4), 99–106.

[CR45] Kaplan-Berkley S (2022). Digital tools and streaming media converge to inspire social interactions of generation alpha. International Journal of Early Childhood.

[CR46] Keirl S, Williams PJ, Jones PJ, Buntting C (2015). ‘Seeing’ and ‘interpreting’ the human-technology phenomenon. The future of technology education.

[CR47] Kermani H, Aldemir J (2015). Preparing children for success: Integrating science, math, and technology in early childhood classroom. Early Child Development and Care.

[CR48] Lindeman S, Svensson M, Enochsson AB (2021). Digitalisation in early childhood education: A domestication theoretical perspective on teachers’ experiences. Education and Information Technologies.

[CR49] Lyons C, Tredwell C (2015). Steps to implementing technology in inclusive early childhood programs. Computers in the Schools.

[CR50] Marsh J, Plowman L, Yamada-Rice D, Bishop J, Lahmar J, Scott F (2018). Play and creativity in young children's use of apps. British Journal of Educational Technology.

[CR51] Maxwell J (1992). Understanding and validity in qualitative research. Harvard Educational Review.

[CR52] McFadden A, Thomas K (2016). Parent perspectives on the implementation of a digital documentation portal in an early learning centre. Australasian Journal of Early Childhood.

[CR53] Murcia K, Campbell C, Aranda G (2018). Trends in early childhood education practice and professional learning with digital technologies. Pedagogika.

[CR54] Neumann MM (2018). Using tablets and apps to enhance emergent literacy skills in young children. Early Childhood Research Quarterly.

[CR55] OECD (2018). Engaging young children: Lessons from research about quality in early childhood education and care. OECD.

[CR56] Parette HP, Quesenberry AC, Blum C (2010). Missing the boat with technology usage in early childhood settings: A 21st century view of developmentally appropriate practice. Early Childhood Education Journal.

[CR57] Parnell WA, Bartlett J (2012). iDocument: How smartphones and tablets are changing documentation in preschool and primary classrooms. Young Children.

[CR58] Penman R (2014). E-portfolios: Connecting parents, whanau and educators in kindergarten communities. Early Education.

[CR59] Plumb, M., & Kautz, K. (2014). Reconfiguring early childhood education and care: A sociomaterial analysis of IT appropriation. In B. Doolin, E. Lamprou, N. Mitev, & L. McLeod (Eds.), *Information systems and global assemblages: (Re)configuring actors, artefacts, organizations* (pp. 30–47). Springer.

[CR60] Roll I, Wylie R (2016). Evolution and revolution in artificial intelligence in education. International Journal of Artificial Intelligence in Education.

[CR61] Romero-Tena R, Barragán-Sánchez R, Llorente-Cejudo C (2020). The challenge of initial training for early childhood teachers. A cross sectional study of their digital competences. Sustainability.

[CR62] Saitis C, Saiti A (2018). Initiation of educators into educational management secrets.

[CR63] Sinclair N, Iliada E, Mulligan J, Anderson A, Baccaglini-Frank A, Benz C (2018). Time, immersion and articulation: Digital technology for early childhood mathematics. Contemporary research and perspectives on early childhood mathematics education.

[CR64] Slot, P. (2018). "*Structural characteristics and process quality in early childhood education and care: A literature review*", OECD Education Working Papers, No. 176, OECD Publishing. 10.1787/edaf3793-en.

[CR65] Sosa Díaz MJ (2021). Emergency remote education, family support and the digital divide in the context of the COVID-19 lockdown. International Journal of Environmental Research and Public Health.

[CR25] Stamopoulos, E., & Barblett, L. (2018). Early childhood leadership in action: Evidence-based approaches for effective practice. Allen & Unwin.

[CR66] Stevenson I (2008). Tool, tutor, environment or resource: Exploring metaphors for digital technology and pedagogy using activity theory. Computers & Education.

[CR67] Stone-MacDonald A, Douglass A (2015). Introducing online training in an early childhood professional development system: Lessons learned in one state. Early Childhood Education Journal.

[CR68] Stratigos T, Fenech M (2021). Early childhood education and care in the app generation: Digital documentation, assessment for learning and parent communication. Australasian Journal of Early Childhood.

[CR69] Su, J., & Yang, W. (2022). Artificial intelligence in early childhood education: A scoping review. *Computers and Education: Artificial Intelligence*, 100049.

[CR70] Thorpe J, Hansen J, Danby S, Zaki FM, Grant S, Houen S, Davidson C, Given LM (2015). Digital access to knowledge in the preschool classroom: Reports from Australia. Early Childhood Research Quarterly.

[CR71] Tondeur J, Aesaert K, Pynoo B, Braak JV, Fraeyman N, Erstad O (2017). Developing a validated instrument to measure preservice teachers’ ICT competences: Meeting the demands of the 21st century. British Journal of Educational Technology.

[CR72] Tout K (2013). Look to the stars: Future directions for the evaluation of quality rating and improvement systems. Early Education and Development.

[CR73] United Nations Association of Australia. (2021). *The digital divide: Lessons Covid-19 taught us about the digital exclusion of students from low socio-economic backgrounds.* https://www.unaa.org.au/2020/11/15/the-digital-divide-lessons-covid-19-taught-us-about-the-digital-exclusion-of-students-from-low-socio-economic-backgrounds/

[CR74] Vanover C, Hodges O (2015). Teaching data use and school leadership. School Leadership & Management.

[CR75] Warschauer M (2002). Reconceptualizing the digital divide. First Monday.

[CR76] Willis L, Exley B (2018). Using an online social media space to engage parents in student learning in the early-years: Enablers and impediments. Digital Education Review.

[CR77] Wright LE, Bales DW (2018). Online professional development for child care providers: Do they have appropriate access to and comfort with the internet?. Journal of Human Sciences and Extension.

[CR78] Yazejian N, Iruka IU (2015). Associations among tiered quality rating and improvement system supports and quality improvement. Early Childhood Research Quarterly.

[CR79] Yost H, Fan S (2014). Social media technologies for collaboration and communication: Perceptions of childcare professionals and families. Australasian Journal of Early Childhood.

[CR80] Irvine, S. & Farrell, A. (2013). The rise of government in early childhood education and care following the Child Care Act 1972: The lasting legacy of the 1990s in setting the reform agenda for ECEC in Australia. Australasian Journal of Early Childhood, 38(4), 99–106.

